# Early outcomes of total hip arthroplasty using point-of-care manufactured patient-specific instruments: a single university hospital’s initial experience

**DOI:** 10.1186/s12893-023-02281-3

**Published:** 2023-12-08

**Authors:** Hieu Pham Trung, Nang Vo Sy Quyen, Nam Vu Tu, Dung Tran Trung, Toan Duong Dinh

**Affiliations:** 1 Hanoi Medical University, Hanoi, Vietnam; 2 Center for Orthopedics and Sports Medicine, Vinmec Healthcare System, Hanoi, Vietnam; 3 3D Technology in Medicine Center, VinUniversity, Hanoi, Vietnam

**Keywords:** 3D Printing, Total hip arthroplasty, THA, Point-of-care manufacturing, Patient-specific instrument, PSI

## Abstract

**Background:**

The use of 3D-printed Patient-Specific Instruments (PSI) has been investigated to enhance the postoperative functional results in total hip arthroplasty (THA) and has been recognized as an innovative approach for the optimal alignment of hip implant components. Point-of-care production is gradually becoming the norm for PSI manufacturing. The purpose of this article is to assess the accuracy and safety of PSI for total hip arthroplasty performed at the point-of-care in Vietnam.

**Methods:**

34 THA cases were assessed in this prospective study. A template for the size and orientation of the implant and the design of the PSI was generated using data from preoperative 3D computed tomography (CT) scanning of the lower limb. The principal surgeon determined the implants’ position and PSI design directly using the software. The PSI is then produced using a 3D-compatible resin printer in our manufacturing hospital. The PSI, consisting of an acetabulum and a femoral component placed press-fit on the bony surface, guided surgeons to precisely ream the acetabulum and cut the femoral neck according to the pre-planned plane. Postoperative CT scanning was obtained and superimposed onto the 3D model of the implant to evaluate the accuracy of the procedure by comparing the orientation values of the cup and the alignment of the stem between the planned and the actual results. Intra- and postoperative clinical parameters of surgery, including surgical time, intra-operative blood loss, complications, and the first ambulation, were also recorded to evaluate the safety of the surgery.

**Results:**

The preparation for PSI required an average of 3 days. 94% of cup size and 91% of stem size were correctly selected. The mean values of postoperative inclination and anteversion were 44.2° ± 4.1° and 19.2° ± 5.6°, respectively. 64.7% of cases deviated from planned within the ± 5^0^ range and 94.1% within the ± 10° range. There was no significant statistical difference between the planned and the achieved values of stem anteversion, osteotomy height, and leg length discrepancy (p > 0.05). The average surgical time was 82.5° ± 10.8 min, and the intraoperative blood loss was estimated at 317.7° ± 57.6 ml. 64.7% of patients could walk on the day of surgery. There were no complications reported.

**Conclusions:**

The point-of-care manufactured PSI is a useful solution for improving the accuracy of total hip arthroplasty surgery, especially in restoring implant orientation and reducing leg length discrepancy. However, long-term clinical follow-up evaluation is needed to confirm the efficacy and safety of this approach.

## Background

Total hip replacement has undergone over a hundred years of improvement and is now one of the most successful and cost-effective procedures widely used all over the world. However, specific complications, including joint dislocation, early loosening, pain, and limited range of motion, persist. The main cause of the aforementioned problem is believed to lie in the improper positioning or suboptimal selection of the implant size, [1] which is largely attributable to the inferior accuracy of 2-dimensional (2D) imaging when compared to 3-dimensional (3D) imaging in THA templating and the lack of precise localization devices during the surgery. Inaccuracy of freehand cup positioning during total hip arthroplasty can lead to more than half of surgeries performed by experienced surgeons being misplaced [2, 3]. The advancements in diagnostic imaging techniques, such as CT scanners, magnetic resonance imaging (MRI), and 3D technology, play an important role in increasing the accuracy of the surgery. Besides, several modern assistive methods have emerged, including computer navigation systems, robotic surgery, and 3D Patient-Specific Instruments (PSI), which also add to higher accuracy and safer and less invasive surgical procedures [4].

Among those technologies, one notable solution that has shown promise in addressing the complications of THA is the use of Patient-Specific Instruments (PSI). The 3D-printed PSI is a personalized navigation technology that provides 3D preoperative planning using the data from the patient’s preoperative CT scan. By tailoring the surgical approach to the individual patient’s anatomy, PSI was designed to enable a perfect fit on the patient’s bone model, hence assisting the surgeon in accurately determining the placement of implant components. Research indicates that the implementation of PSI during THA helps optimize the surgical accuracy of component positioning, leading to improved patient outcomes. Also, this approach provides a helpful and safe measure for surgeons in handling complicated bone and joint deformities and cases at high risk of joint component misplacement [5, 6]. Surgeons who solely rely on mechanical positioning devices or adhere to Lewinnek’s safety range for cup positioning may encounter difficulties or errors, which PSI can help mitigate [7, 8].

Nevertheless, the dependence on commercial PSI products can potentially lead to an increase in the costs and waiting times for the surgery. 3D printing technology is the ideal solution to this problem. Hospitals can now produce their own 3D-printed models on-site [9]. This approach, known as “point-of-care” manufacturing, offers a more customized and timely treatment option for various conditions and overcomes the challenges of space limitations or high installation costs [10]. Also, surgeons involved directly in the PSI design process contribute to the enhancement of the procedure’s safety and precision. To further investigate the accuracy, effectiveness, and safety of utilizing 3D-printed PSI with direct surgeon involvement in the production process, a study was conducted. This research aims to assess how PSI, when integrated into THA procedures, can help mitigate complications and enhance patient outcomes.

## Material and method

### Patient selection

A prospective cross-sectional study was conducted. In total, 34 patients who underwent unilateral primary cementless total hip arthroplasty at Vinmec International Hospital in Hanoi, Vietnam, between April 2022 and May 2023 were included in the study. These patients had either avascular necrosis of the femoral head, a femoral neck fracture, or hip osteoarthritis. Patients with pelvic morphological deformities or significant bone defects requiring the use of augments were excluded from the study.

All patients who agreed to participate in this study were required to follow the protocol of pre- and post-operative CT scans, CT-based templating in preoperative planning, and utilization of PSI during surgical procedures.

The ethics approval was obtained from the Institutional Ethical Review Board of Hanoi Medical University before the study began.

Fourteen of the 34 cases included in this study were previously reported in Vietnamese as a preliminary analysis covering the first phase of this prospective study [33].

### Pre-operative planning

The preoperative planning process involved gathering CT scans from the patients and processing the images using specialized software, determining the expected size and placement of the acetabular cup, as well as the expected size and placement of the stem.

**Fig. 1:**
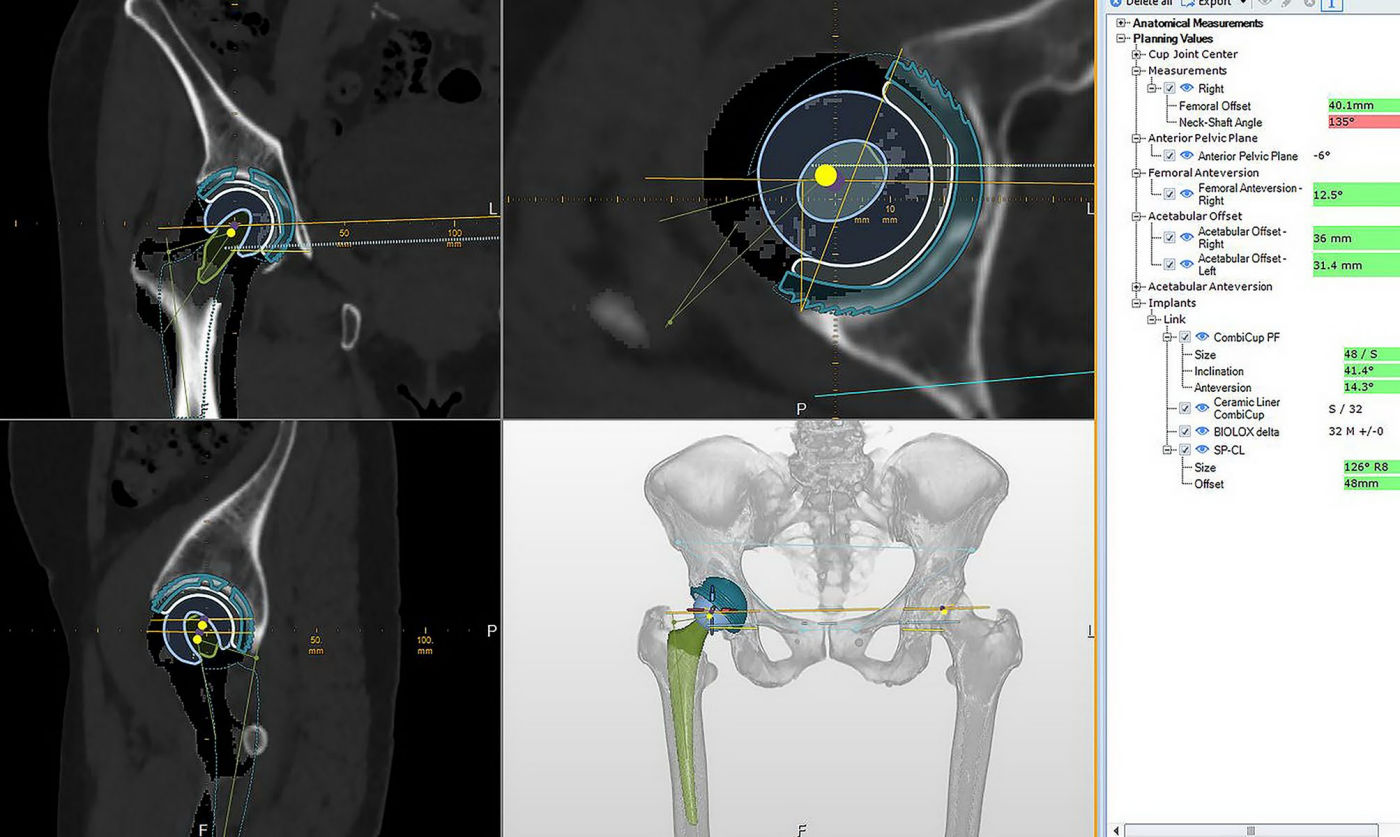
Hip joint implants measurements on the surgical templating software parameters that can be evaluated include anatomical indices of the hip joint, cup and stem size and orientation

At first, patients underwent long-leg supine CT imaging using the Revolution CT 512-slice scanner (G.E., USA), tracking from the iliac crest over the bilateral femoral condyles with a slice thickness of either 0.5 or 0.8 mm to measure the hip joint parameters pre- and post-operation. The imaging data was calibrated and encoded into DICOM format using the Centricity system (G.E., USA). In this step, the radiologist created a series of denoised images and increased the surface detail of the bone tissues. This image series was then imported to MediCAD Hip 3D version 2.0 software (Hectec GmbH, Germany) to form the 3D bone image, enabling 3D digital implant planning and capable of measuring most hip arthroplasty parameters. The template steps are shown in Fig. 1 and clearly described below.

The expected size and the positioning of the acetabular cup were determined during the templating phase. To measure the cup size and design PSI in the next steps, the DICOM files were imported into Mimics and Medicad software and applied coordinates in three dimensions: x, y, and z. These coordinates serve as landmarks for the acetabular cup plane and other bony structures, as well as aid in locating the centers of rotation on both sides. After marking the anatomical landmarks and segmenting separately the pelvis and femur, we proceeded to select the implant size and determine the implant placement in the implant module of the software. The acetabular component is positioned based on the radiographic inclination angle, radiographic anteversion angle, and hip rotation center. The inclination and anteversion were determined based on the radiographic angle definition proposed by Murray [11]. The cup orientation was determined in 3 steps, with the following priorities in turn: The first step is fitting the rotation center by the symmetric method relative to the body’s midline with minimal deviation (within 2 mm) in each direction; the second is achieving optimal acetabular cup coverage with minimally 70% of the cup surface in contact with subchondral bone, as referenced in the study by Wu, and at this step, we determined the size of the cup component that fits the patients the most; [12] and last, the cup was rotated to ensure maximum contact between the anterior and posterior edges of the cup and the rims of the acetabulum while accounting for any deformities caused by bone spurs. The surgeons could adjust the cup to fit within the safe zone defined by Lewinnek in this step (40^0^±10^0^ for inclination and 15^0^±10^0^ for anteversion), [7] but meeting these criteria is not mandatory. In situations where the cup position falls outside the safe zone but meets the requirements for the center of rotation and bone coverage, surgeons still prioritize the orientation option of meeting the first two criteria.

The femoral stem templating included determining the position of the stem and choosing the expected stem size. The first step is stem orientation. The stem anteversion is determined by rotating the stem neck axis parallel to the original femoral neck axis (the original anteversion is defined on Medicad as the angle between the axis passing through the center of the femoral neck and the posterior condyle axis). The second step is to define the stem axis. In this study, researchers only measured the stem axis alignment on the coronal plane to classify the axis alignment as central (the stem axis is oriented toward the central axis of the medullary cavity), varus or valgus (the stem axis is oriented toward the medial wall and vice versa). The predicted stem size needs to ensure the restoration of anatomical femoral offset (the stem offset of the selected implant size must be equal to or greater than the anatomical femoral offset) and achieve a maximum contact area of 80% between the stem surface and the proximal femoral medullary cavity. In this study, only the anatomical stem design (SpCL stem from Link, Germany) was used with a press fit design according to the shape of the femur medulla, so it was easy to achieve the above criteria. The above procedures enabled the selection of the suitable stem size and the measurement of the stem depth in the femur, which informed the calculation of the needed offset of the head implant and the expected leg length discrepancy after the surgery. The choice of stem size and orientation also aimed to minimize the difference in length between the two legs. In the final step, after placing the stem, a resection tool from Medicad was used to create a femoral neck cut plane that intersected the boundary between the neck and body of the stem. The coordinates of this plane were used in the subsequent PSI design step. The femoral neck osteotomy plan is set high from the lesser trochanter (mm) to match the desired orientation and stem fit.

### PSI design and operative procedure

A PSI with two components – a femoral PSI guide and an acetabular guide – was designed based on using the Mimics program version 23.0 (Materialise, Belgium).

**Fig. 2:**
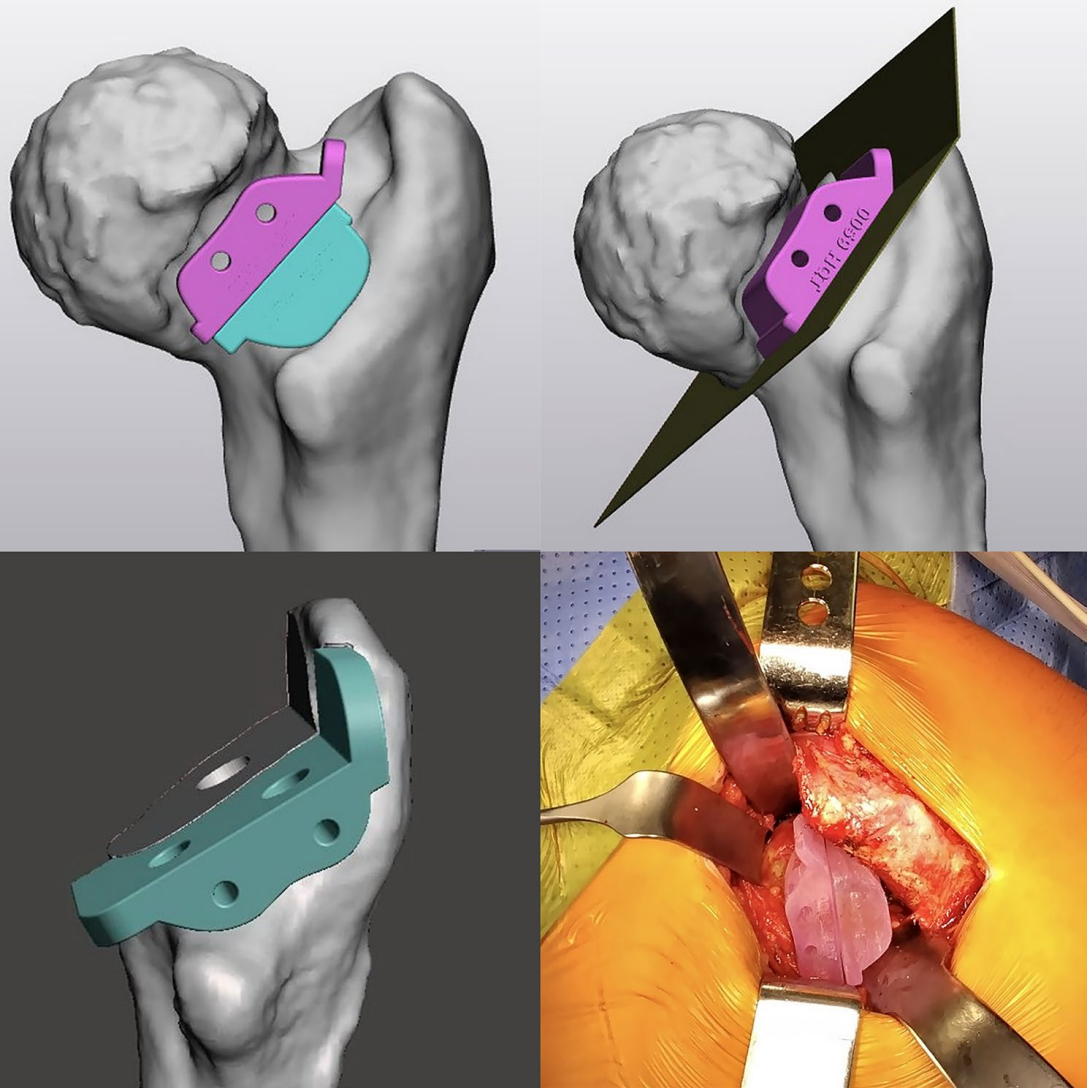
Design and intra-operative use of the femoral neck cutting guide. The PSI has a slotted shape designed to help define the femoral neck cut plane and the lateral edge to create a diagonal orientation to determine stem anteversion

The objective of the femoral PSI is to outline the plane of the femoral neck cut intraoperatively and to determine the stem anteversion based on the templated data. The femoral PSI guide is designed to fit snugly onto the anatomical site of the femoral neck. It is secured to the bone using three 2 mm drill holes. The cutting guide also features a 2 mm-wide groove on the tray (cut plane) to guide the oscillating saw during the cutting process as it templates. In cases where there is a fractured femoral neck or difficulties in exposing the femoral head, the cutting guide can be customized into two separate components. The outer edge of this PSI component is designed to be a diagonal line that helps the surgeon direct the stem neck parallel to help achieve the planned stem anteversion (Fig. 2).

**Fig. 3:**
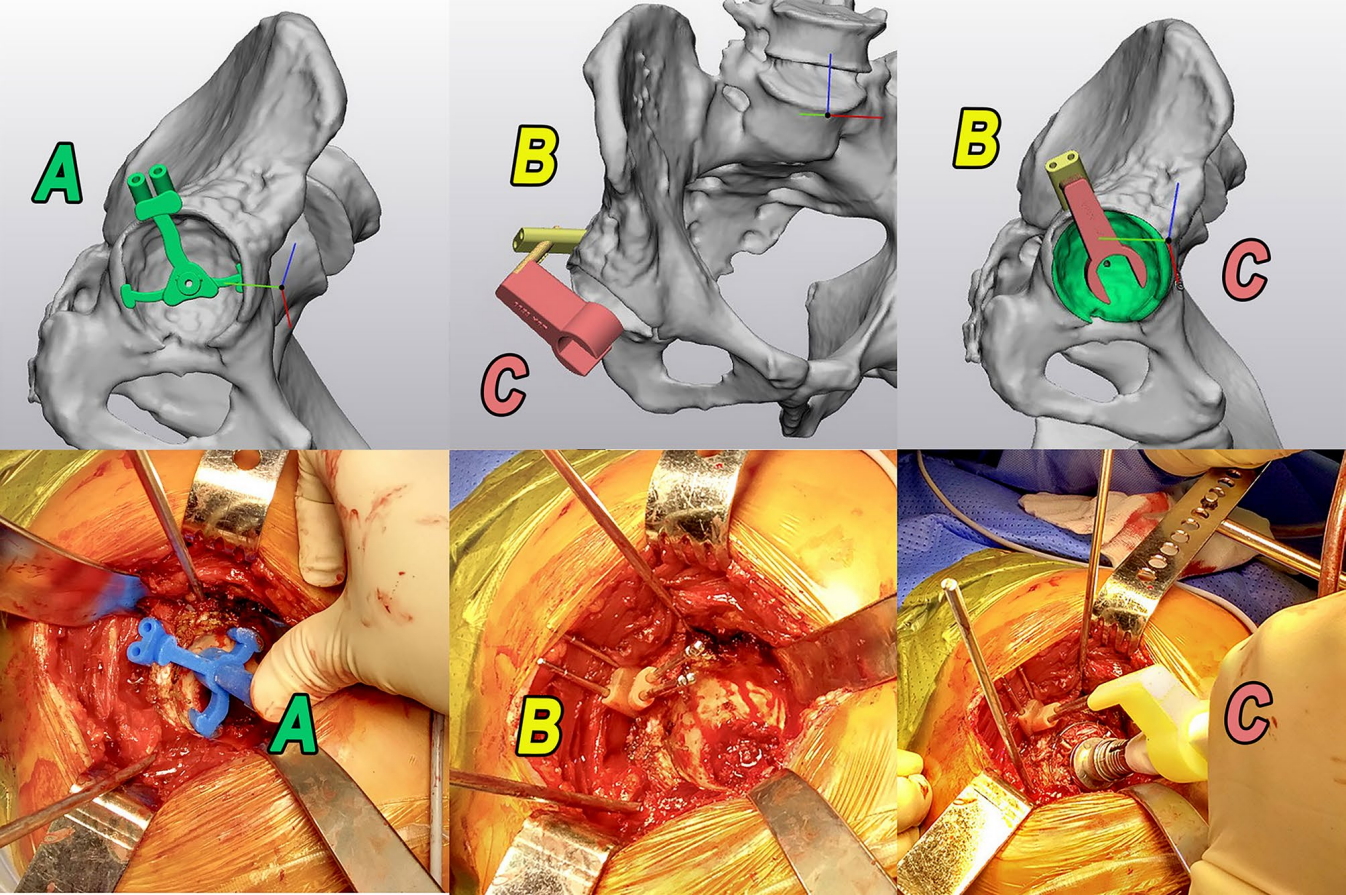
Design and intra-operative use of the Acetabular cup PSI. **A**: Part A is designed to be pressed fit to the acetabulum joint surface and locates 2 pins marked on the superior-posterior rim of the acetabulum; **B**: Part B is designed to be placed on the 2 pins marked by part A, and helps to attach 2 guiding pins that connect to part C; **C**: Part C is designed to connect with guiding pins to accurately orient the reamer handle along the center cup axis

The acetabular PSI guides are constraint instruments with all cup-setting steps. The cup positioning PSI consists of three parts, which are used sequentially during the surgery as follows: (1) Part A is a complex-shaped device that has a surface that interfaces with the articular surface of the acetabular fossa (we made an offset 0.5 mm thick for this surface of design to compensate for deviation by the cartilage, which cannot be observed on a CT scanner). On the posterior edge of part A, there is a hook that attaches to the superior-posterior rim of the acetabulum to prevent PSI from displacing. This hook has 2 holes for marking the position of 2 × 65 mm pins. (2) After putting 2 pins in, remove part A and insert part B, which is a flat cylindrical block, at the same marked positions on the rim of the acetabulum. There are two other holes on the body of Part B to attach two 2 × 100 mm guiding pins for the direction of the reamer handle. The direction of these two pins is parallel to the perpendicular passing through the actual cup plane (cup center axis). (3) Part C is designed with a part of the mount attached to the reamer handle and a part of the “scope”. The height of the connection between the “scope” and the fixed mount is the distance between the two guiding pins and the cup center axis. At the end of the “scope”, there are two holes to match the guiding pins of Part B (Fig. 3). The surgeon proceeds with reaming with the ream direction always locked in the direction of 2 guide pins and the axis of the reamer handle has been set to coincide with the cup center axis. Starting from a small size, step by step, to the expected size of the cup, remove all the surface cartilage to expose the subchondral bone. The actual cup handle is also attached to part C, and the putting direction is also determined by the 2 pins guides above. The actual cup is securely fixed with a press-fit technique and may be reinforced with 1–2 screws into the acetabulum to prevent rotation.

The PSIs were printed by a 3D printer from SprintRay (USA) using FDA-accredited biocompatible resin materials. The printing process followed the quality management principles of ISO 9001:2015 and was sterilized by the Sterrad Sterilization System.

The hip implant system used in this surgery was from Link (Hamburg, Germany). It included Combi cups in various sizes (ranging from 46 to 58 mm) with ceramic liners, SpCl stems in sizes 4 to 10, and ceramic heads in sizes 32 or 36 mm, with offset options of -4, 0, or + 4 mm. All surgeries were performed by the same senior surgeon (HPT) using a consistent surgical technique for total hip arthroplasty through a posterolateral approach.

### Intra- and post-operative assessment

Clinical data were recorded during and after surgery to assess the safety of the procedure, including estimated blood loss (the amount of blood recorded in the suction bottle), surgical time, the occurrence of complications (fractures, dislocations), and the time when patients were able to initiate walking with the assistance of a walking frame (criteria for walking included satisfactory pain control, independent sitting and standing without support, adequate lower limb muscle strength, and absence of any postoperative complications).

**Fig. 4:**
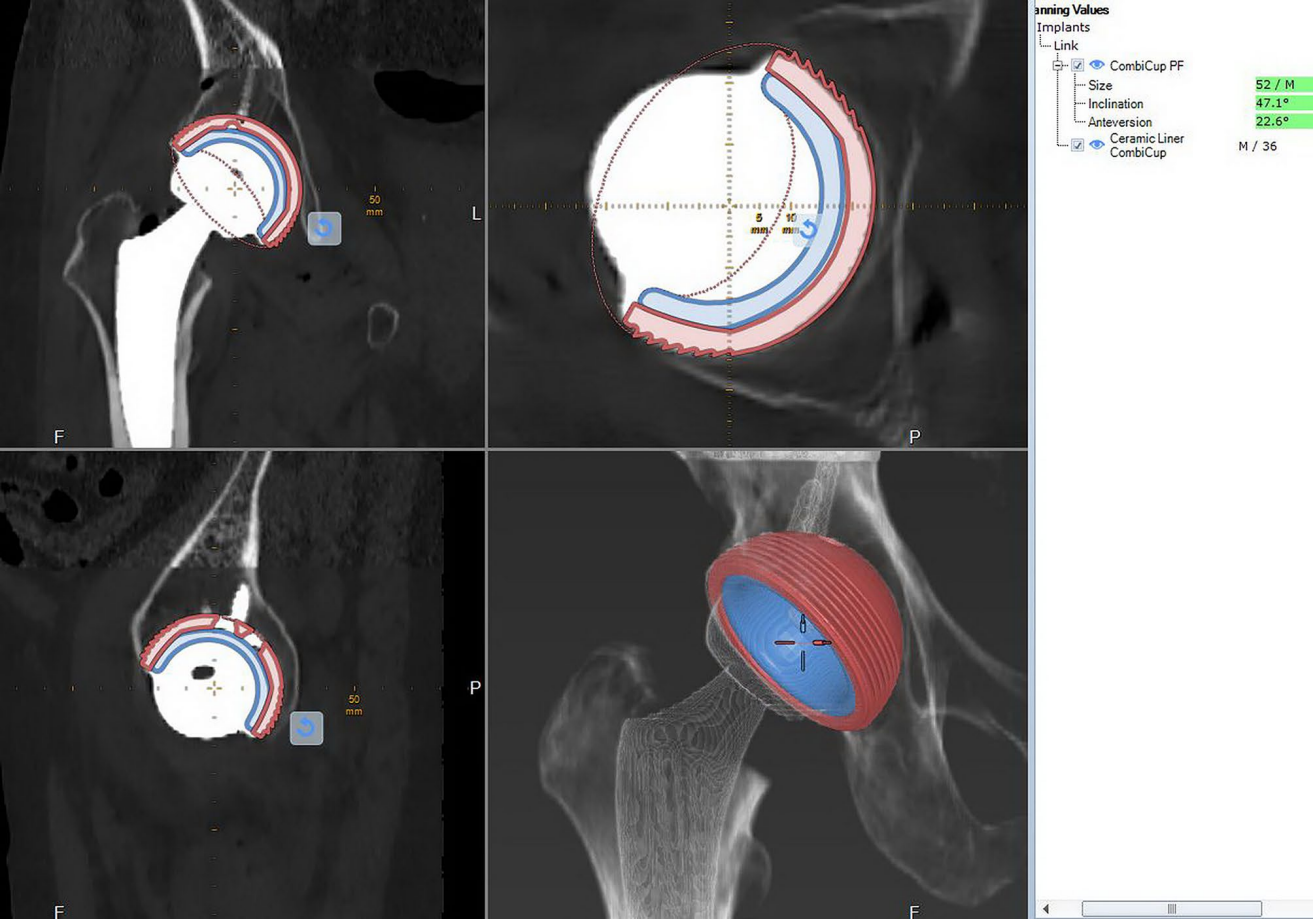
Evaluation of cup orientation on post-operative CT scan by Medicad. Using the method of overlaying the image of implant data available in the software onto the actual implant, it is possible to measure the postoperative indicators and compare them with the templating index

Patients were required to take a CT scan with metal artifact reduction five days after surgery. The data was imported into the Medicad software for further evaluation. Using the existing 3D model data of the implant components in the software, the actual implant positions were superimposed, allowing for the assessment of the achieved acetabular cup orientation, hip rotation center, stem anteversion, and stem axis alignment. Additionally, the actual leg length discrepancy and the actual femoral neck osteotomy height were measured. The above steps are performed similarly to the preoperative measurement process. These results were compared to the expected values obtained from the preoperative templating data (Fig. 4).

The accuracy of the use of PSI was assessed by the discrepancies between preoperatively planned data and those achieved postoperatively. For acetabular cup positioning assessment, cup inclination and anteversion were used as referred indexes. Besides, the author used Lewinnek’s safe zone as the other clinical data for the accuracy assessment. For stem positioning assessment, stem anteversion, neck resection height, and leg length discrepancy are three key indices.

The effectiveness of this method was assessed by the early mobilization of the patients.

The safety of utilizing hip 3D-printed PSI with the direct involvement of surgeons in the production process was assessed by the intraoperative blood loss and occurrence of complications after surgery.

The collected data in the study were entered and processed using SPSS software version 23.0 (IBM, USA). Descriptive statistics (mean, standard deviations, percentage) were used to summarize and describe the demographic and clinical characteristics of the patients in this study. Inferential statistics (Paired Sample t-test, Wilcoxon signed-rank test) were used to compare the preoperatively planned and postoperatively achieved data. A p-value equal to or less than 0.05 is considered statistically significant.

The assumption of normality was checked by the Shapiro-Wilk test for choosing further inferential statistical tests. A parametric test (t-test) was used where a normal distribution is assumed (inclination angle, anteversion angle). A non-parametric test (Wilcoxon signed rank) was applied when the normal distribution was violated.

## Results

### Patients’ demographics

A total of 34 patients participated in this study. The patients’ demographics are reported in Table 1.

**Table 1:** Demographic characteristic of Patients (N = 34)

Characteristics	N	%
Age (years)	Mean ± SD: 55.8 ± 16.6 Min-Max: 17–83	
Gender		
Male	18	52.9
Female	16	47.1
Surgical Side		
Left	12	35.3
Right	22	64.7
Diagnosis		
Avascular necrosis	13	38.2
Femoral neck fracture	8	23.5
Osteoarthritis	13	38.2

### Technical details of the 3D printing project

The preparation for PSI required an average of three days. PSI was designed and manufactured in the hospital by the same team of surgeons. The size of the acetabular cup matched that in the preoperative plan in 94% of cases, and the femoral stem size matched what was planned preoperatively in 91% of cases.

The radiological assessment results are presented in Table 2.

**Fig. 5:**
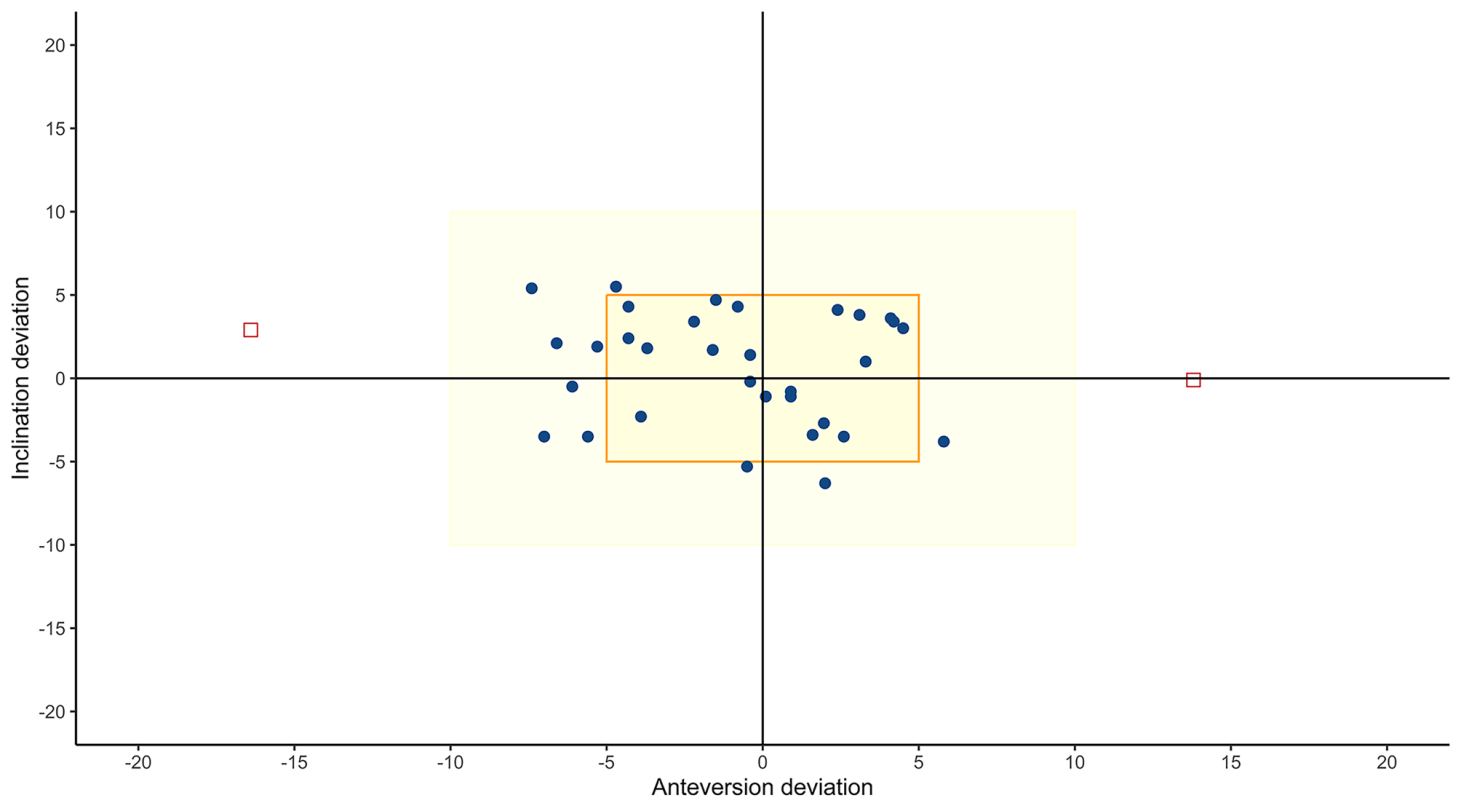
Scatter plot of achieved cup orientation within 5^0^ and 10^0^ range of deviation from template

In 24 cases, accounting for 70.6% of the cases, the orientation of the acetabular cup was reported to stay within Lewinnek’s safe zone. Among them, 4 cases (11.8%) exhibited inclination and anteversion within the range of 40–45 degrees and 15–20 degrees, respectively (this is a common range determined by the mechanical devices that companies provide surgeons with for use in their toolbox). There were no cases in which the acetabular cup was orientted entirely outside Lewinnek’s safe zone in both inclination and anteversion. Regarding the proportion of cases that achieved acetabular component orientation (inclination and anteversion angle) with a deviation of 5 degrees to 10 degrees from the template illustrated in Figs. 5 and 64.7% were within the ± 5^0^ range and 94.1% were within the ± 10^0^ range, comparing the planned and actual achieved values.

The stem axis was aligned with the center in 30 cases (88.2%). There were 3 cases that exhibited varus alignment, accounting for 8.8%. There was 1 hip defined as valgus alignment.

The clinical indices are presented in Table 3. These clinical indices did not significantly differ among patients with different demographic characteristics. 64.7% of patients could walk on the day of surgery. There were no cases of complications (fracture, implant dislocation, limited range of motion) observed during and after surgery in the first 6 weeks.

**Table 2:** Postoperative radiographic assessment (N = 34)

Value		Mean	Min	Max	P-value	Deviation
Cup Inclination(degrees)	Planned	44.9 ± 3.2	38.8	50.9	0.22_*t*_	0.7 ± 3.3
	Achieved	44.2 ± 4.1	36.7	51.8		
Cup Anteversion(degrees)	Planned	18.3 ± 4.8	6.5	28.1	0.28 _*t*_	0.9 ± 5.2
	Achieved	19.2 ± 5.6	7.3	29.4		
Stem Anteversion (degrees)	Planned	18.9 ± 5.5	5.5	28.4	0.14_*w*_	0.8 ± 3.9
	Achieved	18.1 ± 3.8	12.5	25		
Neck resection height (mm)	Planned	11.5 ± 2.8	6.3	19.5	0.11 _*w*_	0.8 ± 2.5
	Achieved	12.3 ± 2.9	7.3	16.8		
Leg length discrepancy (mm)	Planned	1.9 ± 1.2	0.3	4.5	0.45 _*w*_	0.2 ± 1.8
	Achieved	2.1 ± 2.1	0.1	8.3		

Note: t: Paired sample t-test; w: Wilcoxon signed rank

**Table 3:** Postoperative clinical characteristic (N = 34)

Value	Mean ± SD	Min	Max
Surgical Time (minutes)	82.5 ± 10.8	65	105
Intra operative Blood Loss (mL)	317.7 ± 57.6	200	450
First Ambulation (days)	1.5 ± 0.7	1	3

## Discussion

Over the past 15 years, there has been a dramatic rise in surgical-assisted instruments being developed using CT scans and MRI imaging data. Among those, 3D templating and the use of PSI in THA demonstrated superior effects on preoperative planning and postoperative outcomes. The digital data of patients will be segmented to create a customized digital 3D anatomy model, specifically the hip joint and the whole lower limb. Those digital models enable surgeons to observe images in the form of slices, multi-planar reconstructions of CT images, or 3D models that closely resemble reality, thus accurately determining the femoral and acetabular component positioning. Furthermore, 3D software supports calculating the offset of the acetabulum and femur and the precise measurement of leg length discrepancy, thereby minimizing potential complications of the surgery. Lastly, 3D software provides a visualization of the cup orientation and the range of motion of the artificial hip joint in three-dimensional space, offering surgeons a comprehensive understanding of the implant’s orientation and movement for enhanced surgical planning [13, 14, 33].

The main findings of this study highlighted that the 3D printing PSI technique accurately reproduced dynamic planning with regard to the size of the joint components. A study conducted by Mainard demonstrated that the sizing of the stem measured using 3D technology achieved an accuracy of 84%, compared to 68% accuracy when measured using 2D methods. Similarly, the sizing of the cup component was found to be 92% accurate when measured with 3D technology, compared to 87% accuracy with 2D methods [15]. According to Di Laura, the use of 3D templates supports accurately predicting the stem size in 93% of cases and the acetabular cup size in 89% of cases. This significantly reduces the need for hospital inventory reserves by 61% [16]. These findings are similar to those in our study, in which the accuracy in the selection of cup and stem size was 94% and 91% of the cases, respectively. Although our study had a smaller sample size and only utilized one system of hip implants, it still indicates the advantage of using 3D templating in preoperative planning for the anticipated size of the joint components. By accurately predicting the size of the acetabular cup and stem, as well as determining the optimal placement of the components and the osteotomy location, the surgical time can be shortened, and complications such as implant dislocation, early loosening or wear of the joint, periprosthetic fractures, and limb length discrepancies can be controlled [17, 18].

Regarding the accuracy of using PSI for positioning the acetabular component, there is no significant difference in the mean inclination and anteversion values among preoperatively planned data and postoperatively achieved results in this study (44,9^0^ compared with 44.2^0^ and 18.3^0^ compared with 19.2^0^, respectively). Approximately, 64.7% of the cases had an absolute deviation of acetabular component placement within the ± 5^0^ range, and 94.1% of the cases had an absolute deviation of acetabular component placement within the ± 10^0^ range comparing planned data and achieved values. Those findings above are similar to those found by other researchers. Spencer et al. reported the achieved inclination and anteversion values of 41.8^0^ and 25.1^0^, respectively, with an absolute deviation of 3.9^0^ and 3.6^0^, respectively. Approximately 54% of the cases achieved the patient-specific target of inclination and anteversion within the ± 5^0^ range and 91% within the ± 10^0^ range [19]. Findings from Inoue’s study utilizing MRI-based PSI demonstrated that the achieved inclination and anteversion angles were 45.6^0^ and 27.4^0^, respectively, with a deviation of 2.8^0^ and 3.2^0^ degrees from the planned values. Notably, 100% of the inclination measurements fell within the range of ± 10° deviation from the expected values [20]. Ferretti assessed the accuracy of PSI with laser guidance from the OPS system (Corin, UK) and revealed that the achieved inclination and anteversion angles were 38.4^0^ and 18.3^0^, respectively, and in 92% of cases, both inclination and anteversion values were within ± 10° absolute deviation range from the planned values [24]. The findings from this study and others mentioned above indicate that the utilization of PSI point-of-care manufactured, based on the landmarks of the acetabular bone and using the parameters from 3D templating, can ensure accurate determination of the optimal position for the placement of the acetabular component, even in cases with bone deformities. Liang’s study supported this summary [21].

In this study, in 70.6% of the cases, the cups were completely placed within the safe zone of Lewinnek. Given the low rate (11.8%) of cup orientations within the tolerable range of mechanical aligners, surgeons may consider not being overly reliant on available tools but with limited recalibration capabilities. And that provides an opportunity for future research to expand, especially with controlled studies. While the Lewinnek safe zone is widely used in clinical practice in many countries and is sometimes regarded as the gold standard for predicting stability after THA, the researchers acknowledged that there has been considerable debate in the literature concerning this issue. However, the researchers emphasized the need for a reference interval to evaluate the patient outcome in this study to align with clinical practice in Vietnam. As the initial requirements of cup orientation were met, we were willing to accept certain cases of cups outside the reference safe zone. Although there is controversy about the optimal position for a cup with Lewinnek safe zone, it is sometimes a good thing that a newly developed technique produces cup orientation results within a range familiar to many surgeons because it helps these surgeons have more confidence and acceptability to use this product. It is essential to note that for optimal results in terms of hip joint motion range and implant survival, it is crucial to consider the relationship between the positioning of the cup and the femoral offset or stem anteversion. However, these factors were not evaluated in our study. To establish precise correlations, further research will be required, combining computer simulations with clinical outcomes. This will help define safe ranges for implant component placement.

About the PSI for femoral component positioning, the height of the femoral neck osteotomy and the leg length discrepancy achieved postoperatively are similar to those planned preoperatively. The achieved femoral neck osteotomy height varied from the predicted 11.5 to 12.3 mm in actuality. The average leg length discrepancy in this study was 2.1 mm, and the difference in the limb length discrepancy between the planned and achieved was 0.2 mm. We found the similarity of this figure in the study of Mishra, which showed that the use of PSI can achieve a leg length discrepancy between the planned and achieved measurements as low as 0.15 mm [22]. Ferretti’s findings demonstrate that the leg length discrepancy was accurately placed according to the plan, with differences of 2.5 mm expected versus 2.4 mm observed, respectively, similar to this study [23]. Clinically, most patients did not perceive any discomfort during movement. A finding from a study by Hassani that the restoration of balanced leg length reached 88% with an average discrepancy between the two legs of only 0.3 mm pointed out that the finding is understandable [24].

PSI used for femoral bone osteotomy is relatively easy to use (as it involves less interference with soft tissue around the cutting side) while still providing high accuracy. The precise placement of the femoral neck osteotomy plays an important role in establishing reference points for achieving proper leg length, anteversion, and stem axis alignment during stem insertion. Dimitriou’s study showed that femoral neck cut level was correlated with stem anteversion and stem axis alignment [25]. It might be a good idea to use PSI to determine the neck section plane that will help support the correct placement of the stem as templated preoperatively. According to the results in Table 2, the stem anteversion angle among patients recorded after surgery is slightly different from the planned stem anteversion angle. However, the difference was minimal and not statistically significant (18.90 and 18.10). Most stem axis alignments achieved center or varus orientation, with one instance of valgus placement. In this study, all stems were placed press-fit, with the majority having a center-directed stem axis, maximizing the longevity of the stem, and minimizing complications such as thigh pain during ambulation. However, the study by Belzunce figured out that correctly placing the stem anteversion following the anatomy neck axis for cementless stems is challenging, as there is always a difference between planning and reality when evaluating all 6 degrees of freedom (3 for orientation and 3 for position) [26]. According to Hirata’s research, restoring the stem anteversion to match the template can be challenging when the difference between the stem anteversion and the native anteversion is up to 9.8^0^ [27]. However, it is worth noting that Hirata’s study used a taper wedge stem design, which is different from the anatomical stem design we used. Our stem design is more compatible with the femur medulla morphology, which could lead to different results. Nonetheless, these findings can help us enhance the PSI design for each type of implant in future.

The average surgical time when using PSI is longer compared to conventional methods in some studies [22, 28, 29]. In Mishra’s study, the THA surgical time while using PSI was reported as 99.39 min, which was longer than that of the conventional surgery group (surgical time of 92.33 min) [22]. Xing’s et al. reported that the average surgical time was 138.4 min in the PSI group, longer than that in the group of patients receiving conventional surgery [29]. The results of our study showed a shorter surgical time of 82.5 min compared to the above publications, which could be attributed to the majority of patients in our study using smaller implant sizes and performing surgery on patients without severe deformities, leading to a faster preparation time. The longer surgical time of the PSI-involved surgery compared to conventional surgery is supposed to arise from adding the time for PSI application during the operation. However, the differences are not significant. Xiao (2020) figured out that PSI did not prolong the surgical time [6]. According to Spencer, the preparation steps for PSI take approximately 3–5 min [19].

The average amount of blood loss among patients in our study was estimated at 317.7 mL. The study by Xing recorded this figure at 470.0 ± 134.7 mL in the PSI group [29]. The lower intraoperative blood loss among patients in our study compared to Xing’s study can be explained by the shorter operative time (82.5 ± 10.8 min in this study compared to 138.4 ± 32.2 min in Xing’s study). Other factors might also contribute to this difference, including the method used to measure the blood loss, the type of surgery, and the surgeon’s experience. It would be preferable if we could compare the amount of blood lost during surgery between a control group using traditional and PSI surgical groups. However, a limitation of our study is that the PSI surgical method is very new in our country, and this study is a pilot study. A further randomized control trial study should be conducted in the future to investigate more about this aspect.

The postoperative complications in this study included revision, dislocation, and fracture. There was no dislocation, revision, or fracture, demonstrating the safety of this surgical method. In addition, patients in this study were able to walk early after surgery (all patients took their first walk within the first three days after surgery, of whom 64.7% could walk on the day of surgery). Previous studies have shown that allowing patients to walk again on the day of hip replacement surgery is beneficial for shortening hospital stays and significantly improving rehabilitation [30, 31]. Previous research illustrated that PSI significantly helped improve the clinical outcomes of patients after THA [29]. This study assessed the early outcomes of THA indicates similar outcome. However, it is important to note that our study was conducted with a small sample size, had a short follow-up period, and had limited clinical outcomes assessed. A longer follow-up period is required to provide more comprehensive images.

Despite listing some advantages above, 3D planning and patient-specific instrument (PSI) utilization in THA still have some significant drawbacks that are gradually being addressed. Firstly, there is a high cost associated with 3D printers, software investment, and the issues of CT scans, making it unaffordable for every facility to implement. Additionally, patients are exposed to higher levels of radiation. Furthermore, the data from CT scans does not accurately assess certain pathologies and abnormalities in cartilage and soft tissues surrounding the joint, maybe leading to inaccuracies in the surgical instruments. Moreover, the time required for designing and printing a set of instruments can sometimes extend to 1–3 days, increasing the waiting time (although it is faster compared to ordering from other commercial companies worldwide, such as OPS from Corin or My Hip from MedActa and there is no need to depend on the mandatory use of implants by these companies). In order to produce a quality PSI product, a team consisting of surgeons, a design engineer, a printer operation engineer, and individuals in research, finance, and product distribution are required. Our research is conducted in a private healthcare system with significant investment in equipment and personnel, serving research and brand development on a non-profit basis. Therefore, we expect the cost of our commercialized products to be around 300 to 400 USD. Through the hub and spoke model, we plan to gradually expand to other surgeons, thus overcoming the disadvantage of requiring large investment funds for product development.

Following this piloting, regarding the knowledge and understanding of the dissection and use of PSI, we highly recommend that senior surgeons participate in the phases of surgical planning, PSI templating, and utilization. Inexperienced surgeons could participate in the procedure as observers or perform surgery using PSI after being trained and under the direct supervision of the senior ones. Research by Jone revealed that the use of PSI allows inexperienced surgeons to achieve the bone cut at the same level of accuracy as expert surgeons piloting sawbone models. However, there is still a lack of evidence about the ease of use of the PSI for inexperienced surgeons in the clinical setting [32]. Especially in THA, the surgeon needs enough experience to expose the many layers of surrounding muscle and soft tissue to perform this procedure correctly.

## Conclusion

The initial clinical results and postoperative 3D CT scan images demonstrate that the use of hip 3D printed PSI in point-of-care manufacturing enables a more accurate selection of implant size, precise restoration of implant orientation, effectively controls leg length discrepancy, and ensures the treatment outcome equivalent safety and efficacy compared to conventional THA methods. This is also a faster and more affordable solution than using commercial products. As a pilot trial, further expansion of surgical cases, further monitoring, and the evaluation of long-term clinical outcomes are necessary to draw accurate conclusions.

## Data Availability

The data are available from Vinmec Times City Hospital. To obtain the data from this study, please contact Mr. Pham Trung Hieu at v.hieutp24@vinmec.com.

## Abbreviations

2D: 2-dimentional
3D: 3-dimentional
CT: Computed Tomography
PSI: Patient-Specific Instrument
ST: Surgical Technician
THA: Total Hip Arthroplasty
