# Correction: Early outcomes of total hip arthroplasty using point-of-care manufactured patient-specific instruments: a single university hospital’s initial experience

**DOI:** 10.1186/s12893-026-04024-6

**Published:** 2026-07-17

**Authors:** Hieu Pham Trung, Nang Vo Sy Quyen, Nam Vu Tu, Dung Tran Trung, Toan Duong Dinh

**Affiliations:** 1 Hanoi Medical University, Hanoi, Vietnam; 2 Center for Orthopedics and Sports Medicine, Vinmec Healthcare System, Hanoi, Vietnam; 3 3D Technology in Medicine Center, VinUniversity, Hanoi, Vietnam

**Correction: BMC Surg 23**,** 369 (2023)**

**https://doi.org/10.1186/s12893-023-02281-3**

In article [1], the author has identified an error.

1. **Nature of the error**
  An earlier preliminary report describing the first 14 cases of the cohort was not cited in the References section of our article. The preliminary report was published in Vietnamese in the Journal of Medical Research (Hanoi Medical University) in January 2023 and constitutes a subset of the 34 cases reported in the BMC Surgery article. The omission was an unintentional citation oversight at the time of manuscript preparation in mid-2023, when the Vietnamese preliminary report had been published only a few months earlier and was not yet widely indexed in international databases.
2. **Proposed corrections**
  - Add a new reference [33] to the References list:
    Pham TH, Vo SQN, Vu TN, Phan KN, Tran TD, Duong DT. Evaluation of the early outcome of total hip replacement surgery using patient-specific instrument. J Med Res. 2023;162(1):147–156. doi:10.52852/tcncyh.v162i1.1348
  - Add the following sentence at the end of the “Patient selection” subsection in Materials and Methods (page 2):
    "Fourteen of the 34 cases included in this study were previously reported in Vietnamese as a preliminary analysis covering the first phase of this prospective study [33]."
  - Add a citation to reference [33] in the introductory paragraph of the Discussion section (page 7), where we discuss the development of CT-based 3D templating.
3. **Justification**
  ICMJE Recommendations (Section IV.B.3 on overlapping publications) require that any prior related publication of the same data be transparently cited, even when the subsequent publication represents a substantial extension. While the BMC Surgery article more than doubles the cohort (from 14 to 34 cases) and introduces an entirely new analytical framework, the 14 cases of the preliminary report remain a subset of the cohort reported, and cross-citation is the standard practice recommended by ICMJE and COPE.
  Second, this matter has been formally raised by the doctoral dissertation committee at Hanoi Medical University, where the first author (Pham Trung Hieu) is currently completing his PhD review.
  A published Author Correction provides clear, public documentation of our commitment to transparency in the publication record.
  The proposed change is minimal in scope: the addition of one reference [33] to the References list, one brief explanatory sentence at the end of the "Patient selection" subsection (page 2), and the insertion of citation [33] into the existing citation “[13, 14]” in the Discussion (page 7). It does not alter the methodology, results, or conclusions of the article. All co-authors have unanimously consented to this correction.
4. **Authors’ consent**
  All Authors (Hieu Pham Trung, Nang Vo Sy Quyen, Nam Vu Tu, Dung Tran Trung, and Toan Duong Dinh) have been informed and have unanimously consented to this correction request.

The original article has been corrected.
